# Glucocorticoids suppress inflammation via the upregulation of negative regulator IRAK-M

**DOI:** 10.1038/ncomms7062

**Published:** 2015-01-14

**Authors:** Masanori Miyata, Ji-Yun Lee, Seiko Susuki-Miyata, Wenzhuo Y. Wang, Haidong Xu, Hirofumi Kai, Koichi S. Kobayashi, Richard A. Flavell, Jian-Dong Li

**Affiliations:** 1 Center for Inflammation, Immunity & Infection, Institute for Biomedical Sciences, Georgia State University, Atlanta, Georgia 30302, USA; 2 Department of Microbiology and Immunology, University of Rochester Medical Center, Rochester, New York 14642, USA; 3 Department of Molecular Medicine, Graduate School of Pharmaceutical Sciences, Kumamoto University, Kumamoto 862-0973, Japan; 4 Department of Microbial Pathogenesis and Immunology, College of Medicine, Texas A&M Health Science Center, College Station, Texas 77843, USA; 5 Department of Immunobiology and Howard Hughes Medical Institute, Yale University School of Medicine, New Haven, Connecticut 06520, USA

## Abstract

Glucocorticoids are among the most commonly used anti-inflammatory agents. Despite the enormous efforts in elucidating the glucocorticoid-mediated anti-inflammatory actions, how glucocorticoids tightly control overactive inflammatory response is not fully understood. Here we show that glucocorticoids suppress bacteria-induced inflammation by enhancing IRAK-M, a central negative regulator of Toll-like receptor signalling. The ability of glucocorticoids to suppress pulmonary inflammation induced by non-typeable *Haemophilus influenzae* is significantly attenuated in IRAK-M-deficient mice. Glucocorticoids improve the survival rate after a lethal non-typeable *Haemophilus influenzae* infection in wild-type mice, but not in IRAK-M-deficient mice. Moreover, we show that glucocorticoids and non-typeable *Haemophilus influenzae* synergistically upregulate IRAK-M expression via mutually and synergistically enhancing p65 and glucocorticoid receptor binding to the IRAK-M promoter. Together, our studies unveil a mechanism by which glucocorticoids tightly control the inflammatory response and host defense via the induction of IRAK-M and may lead to further development of anti-inflammatory therapeutic strategies.

## Abstract (web summary)

Glucocorticoids strongly suppress inflammation. Here the authors show that this suppression is mediated by induction of the negative inflammatory regulator IRAK-M, and demonstrate its important role in host defense against the pneumonia-causative bacterium, non-typeable *Haemophilus influenzae*.

## Graphical abstract

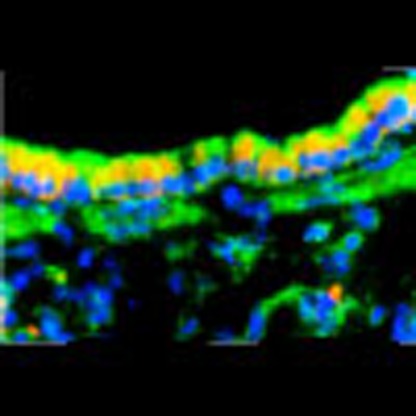

Glucocorticoids (GCs) are the most widely used and most effective treatment to control inflammatory diseases^1^,^2^,^3^. GCs are known to exert their anti-inflammatory effects by binding to glucocorticoid receptors (GRs), leading to the suppression of proinflammatory regulators such as nuclear factor-κB (NF-κB) or activator protein 1 (AP-1) (refs 4, 5). Several mechanisms of action of GR have been reported in the past^6^. First, GR binds to p65 and AP-1 to prevent downstream transcription (tethering). Second, GR binds to the glucocorticoid response element (GRE) to initiate the transcription of anti-inflammatory genes (transactivation). Third, negative GRE has been shown to suppress proinflammatory cytokines (transrepression)^7^,^8^.

The innate immune and inflammatory response is activated by pattern recognition receptors, including Toll-like receptors (TLRs), on recognition of pathogen-associated molecular patterns^9^. Pattern recognition receptors activate a number of downstream molecules such as tumour necrosis factor (TNF) receptor-associated factor 6 (TRAF6), NF-κB essential modulator, inhibitor of NF-κB (IκB) kinase β (IKKβ) and NF-κB to produce proinflammatory cytokines^9^. Myeloid differentiation factor 88 (MyD88) is a critical downstream adaptor molecule of all TLRs, except TLR3, and interleukin-1 (IL-1) receptor (IL-1R) family (IL-1α, IL-1β, IL-18 and IL-33) signalling by recruiting IL-1R-associated kinase 1 (IRAK1), IRAK4 and TRAF6 (refs 9, 10). The studies of MyD88-deficient mice and humans suggest that MyD88 plays a pivotal role in initiating inflammatory responses^11^,^12^.

Negative feedback regulators of inflammation have been recently suggested to play essential roles in tightly controlling inflammatory responses to preserve homeostasis^10^. IRAK-M (also known as IRAK3) is one of the most critical negative feedback regulators of the TLR/IL-1R family signalling via the inhibition of MyD88 and IRAK1/4 activation^13^,^14^,^15^,^16^. IRAK-M is a member of the IRAK family and is composed of three conserved domains. However, it does not have any kinase activity due to the lack of a key aspartate in its kinase domain. Thus, IRAK-M has been thought to be a competitor for IRAK1 in associating with MyD88 and TRAF6 (ref. 13). Indeed, IRAK-M-deficient mice exhibited increased inflammatory response in several models^15^,^16^,^17^,^18^,^19^. IRAK-M expression was initially characterized in monocytes/macrophages^16^,^20^,^21^. Recent studies also demonstrate the expression of IRAK-M in airway epithelial cells^20^,^22^. Induction of IRAK-M expression by a number of inflammatory stimuli has been shown to suppress inflammation in a negative feedback manner in multiple cell types including macrophages and epithelial cells^18^,^19^. However, it is unclear if GCs suppress overactive inflammatory responses via induction of negative feedback regulators such as IRAK-M.

In the present study, we show that GCs synergistically enhances IRAK-M expression induced by non-typeable *Haemophilus influenzae* (NTHi), not only in airway epithelial cells, but also in macrophages. We found that the overexpression of IRAK-M suppresses, whereas IRAK-M depletion enhances, the NTHi-induced expression of proinflammatory mediators. We further found that IRAK-M-deficiency attenuates the ability of dexamethasone (DEX) to suppress pulmonary inflammation induced by NTHi infection. Lethal NTHi infection-caused mortality was improved by DEX treatment in wild type (WT), but not in IRAK-M-deficient mice. Together our results suggest that the induction of IRAK-M by GCs may be critical to suppress overactive pulmonary inflammatory response *in vitro* and *in vivo*. These results thus identified IRAK-M as a novel functional target of GCs.

## Results

### GCs synergistically enhance NTHi-induced IRAK-M expression

**Figure 1:**
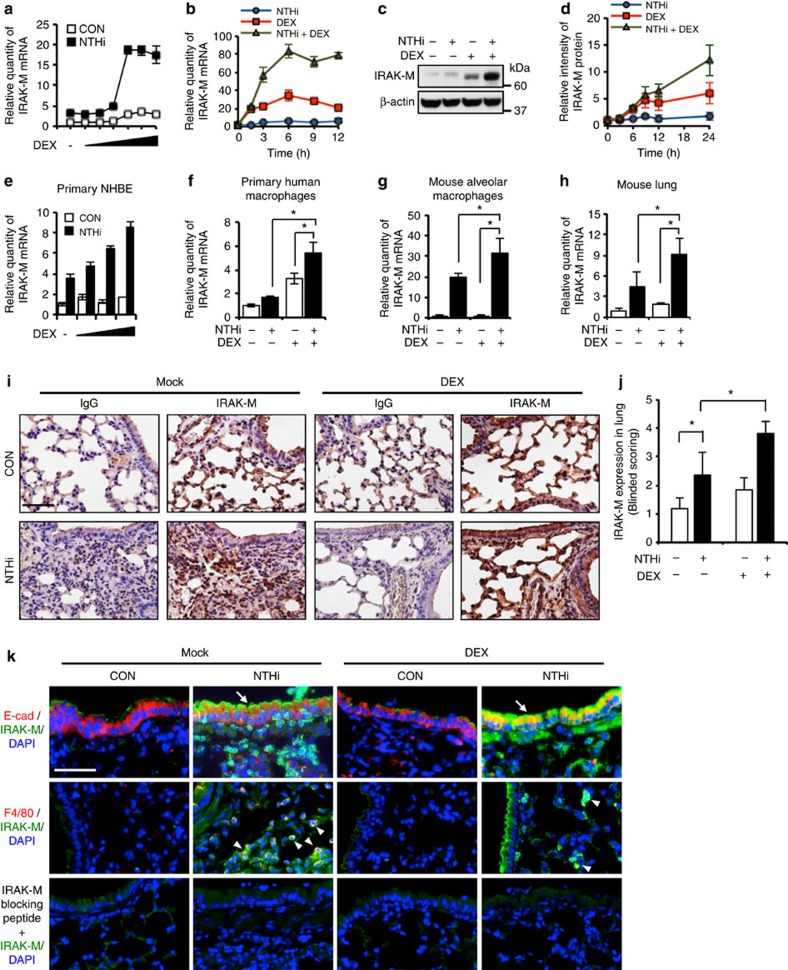
DEX synergistically enhances the NTHi-induced IRAK-M expression *in vitro* and *in vivo*. (**a**) IRAK-M mRNA expression in human bronchial epithelial BEAS-2B cells treated with DEX (0.01, 0.1, 1, 10, 100 or 1,000 nM) for 1 h, followed by the stimulation of NTHi for 5 h. (**b**) IRAK-M mRNA expression in human lung epithelial A549 cells stimulated with DEX (100 nM) for 1 h, followed by NTHi for indicated time. (**c**) A549 cells were treated with DEX (100 nM) for 1 h, followed by NTHi for 12 h. IRAK-M protein was detected by anti-IRAK-M antibody. (**d**) IRAK-M protein expression was quantified from three independent experiments. (**e**) IRAK-M mRNA expression in primary NHBE cells treated with DEX (10, 100 or 1,000 nM) for 1 h and subjected to NTHi stimulation for 5 h. (**f**) Human peripheral blood CD14^+^ monocytes were differentiated to macrophages by culturing them with granulocyte–macrophage colony-stimulating factor for 7 days. IRAK-M mRNA was determined after the macrophages were treated with DEX (100 nM) for 1 h, followed by stimulation of NTHi for 5 h. (**g**) Mice were stimulated with DEX (1 mg kg^−1^, intraperitoneally) for 2 h and intratracheally inoculated with NTHi (1 × 10^7^ c.f.u.) for 24 h. The mRNA expression in alveolar macrophages was assessed by qPCR. (**h**) The IRAK-M mRNA expression in lung was assessed by qPCR. (**i**) Immunostaining of mouse lung with control IgG or anti-IRAK-M antibody by LSAB (Labelled Streptavidin Biotin) staining system (Scale bar, 50 μm; magnification, × 400). (**j**) Treatment-blind observers scored the IRAK-M expression from the histology results. (**k**) The lung sections were stained by using indicated antibodies. The arrows and arrowheads indicate the epithelial cells and macrophages merged with IRAK-M expression, respectively. (Scale bar, 50 μm; magnification, × 400. Data in **a**,**b**,**d**-**h**, *n*=3; **j**, *n*=6) are mean±s.d. *P<0.05; *t-*test.

We sought to determine if GCs regulate the expression of IRAK-M induced by NTHi. Because airway epithelial cells are the front line of defense through initiating inflammatory response, we first examined the effect of GCs on NTHi-induced expression in human respiratory epithelial cells BEAS-2B and A549. DEX synergistically enhanced the NTHi-induced IRAK-M expression at the mRNA level in BEAS-2B and A549 in a dose- and time-dependent manner (Fig. 1a,b and Supplementary Fig. 1a,b). Immunoblot analysis revealed that DEX also synergistically enhanced NTHi-induced IRAK-M expression at the protein level (Fig. 1c,d). Moreover, DEX-mediated synergistic enhancement of NTHi-induced IRAK-M expression is also confirmed in primary normal human bronchial epithelial cells (Fig. 1e). We also examined the effect of GCs on IRAK-M expression in macrophages due to their important role in innate immune responses against bacteria^23^,^24^. As shown in Fig. 1f, the induction of IRAK-M by NTHi and DEX was also observed in primary human monocyte-derived macrophages. Under the same experimental condition, NTHi-induced IL-6 expression was suppressed by DEX (Supplementary Fig. 2). Consistent with these results, DEX also synergistically enhanced the NTHi-induced IRAK-M expression in primary alveolar macrophages of mice (Fig. 1g). Moreover, DEX also synergistically enhanced the NTHi-induced IRAK-M expression at both mRNA and protein levels in lung tissue of mice, as assessed by performing qPCR and immunostaining analyses (Fig. 1h–j). To further determine which cells express IRAK-M in the lung of mice treated with NTHi and DEX, we next performed immunofluorescence double staining using anti-IRAK-M with anti-E-cadherin or anti-F4/80 antibodies, the marker of epithelial cells and macrophages, respectively. As shown in Fig. 1k, NTHi and DEX markedly induced the expression of IRAK-M in both epithelial cells and macrophages. Of note, IRAK-M-specific staining was completely abolished by an IRAK-M-specific blocking peptide (Fig. 1k). Taken together, our data suggest that DEX synergistically enhances NTHi-induced IRAK-M expression at both mRNA and protein levels *in vitro* and *in vivo*.

### IRAK-M negatively regulates the NTHi-induced inflammation

**Figure 2:**
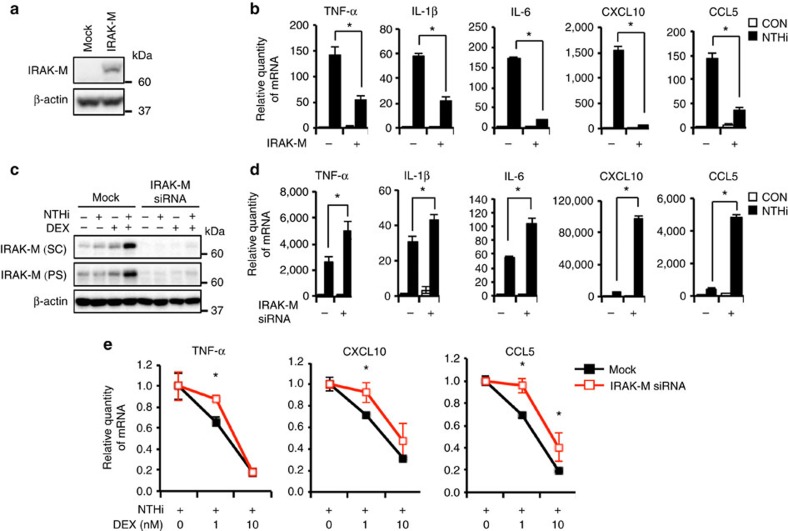
IRAK-M suppresses the NTHi-induced proinflammatory mediator expression. (**a**) The proteins from BEAS-2B cells stably overexpressing IRAK-M and its control pcDNA3.1 (Mock) cells were detected by immunoblot with indicated antibodies. (**b**) The mRNA expression was detected by qPCR after the stable cells were treated with DEX (100 nM) and NTHi. (**c**) BEAS-2B cells were transfected with siRNA control or IRAK-M for 72 h. IRAK-M protein expression was detected by immunoblot with anti-IRAK-M antibodies (SC, Santa Cruz Biotechnology sc-100389; PS, ProSci #2355). (**d**) siRNA-transfected BEAS-2B cells were infected with NTHi followed by qPCR. (**e**) The mRNA level was calculated by following: (NTHi-induced mRNA at each concentration of DEX)/(NTHi-induced mRNA without DEX). Data in **b**,**d**,**e**, *n*=3) are mean±s.d. *P<0.05; *t-*test.

To determine the role of the induction of IRAK-M expression in the NTHi-induced inflammatory responses, we established stable BEAS-2B cells expressing IRAK-M (IRAK-M-stable cells; Fig. 2a). As shown in Fig. 2b, the NTHi-induced expression of TNF-α, IL-1β, IL-6, CXCL10 and CCL5 was significantly inhibited in IRAK-M-stable cells as compared with mock cells. Next we sought to confirm the role of endogenous IRAK-M expression in NTHi-induced expression of proinflammatory mediators by depleting IRAK-M using short interfering RNA (siRNA). Immunoblot analysis using two different anti-IRAK-M antibodies revealed that the siRNA depletion of IRAK-M specifically reduced IRAK-M expression (Fig. 2c). IRAK-M depletion markedly enhanced NTHi-induced expression of TNF-α, IL-1β, IL-6, CXCL10 and CCL5 (Fig. 2d). Moreover, the ability of DEX to suppress these proinflammatory mediators was also significantly attenuated in IRAK-M-depleted cells (Fig. 2e). These data thus provide direct evidence for the role of the induction of IRAK-M expression in NTHi-induced inflammatory responses in the presence of DEX.

### GCs suppress inflammation and improve survival via IRAK-M

**Figure 3:**
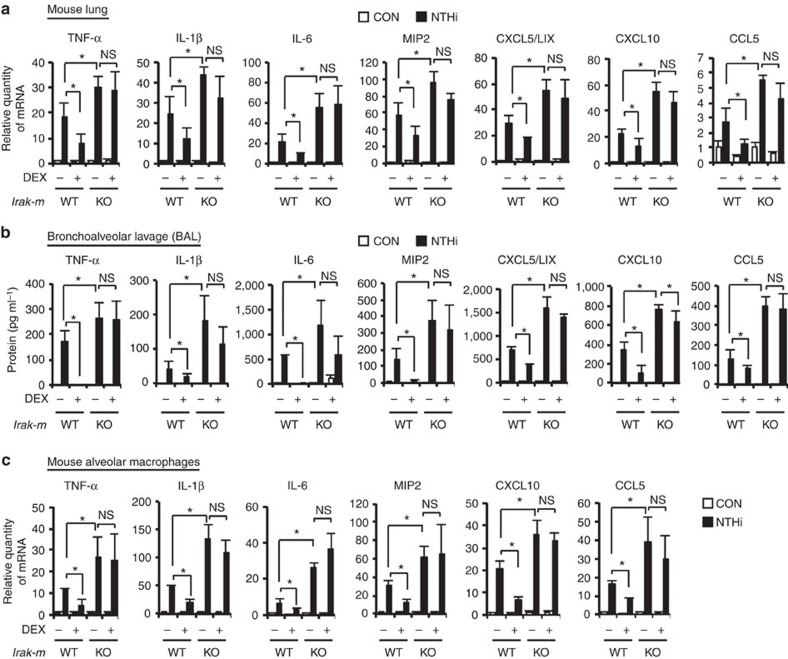
DEX suppresses the NTHi-induced proinflammatory mediator expression in *Irak-m*^*+/+*^ but not in *Irak-m*^−/−^ mice. WT and IRAK-M-deficient mice were inoculated with DEX (1 mg kg^−1^, intraperitoneally) for 2 h, followed by intratracheal inoculation with NTHi (1 × 10^7^ c.f.u. per lung) for 24 h. (**a**) The mRNA expression in the mouse lung tissue was analysed by qPCR. (**b**) The protein level of proinflammatory mediators in BAL fluid was determined by enzyme-linked immunosorbent assay. (**c**) The mRNA expression in alveolar macrophages was analysed by qPCR. Data in **a**,**c**, *n*=3; **b**, *n*=9 are mean±s.d. *P<0.05, NS, non-significant; *t-*test.

**Figure 4:**
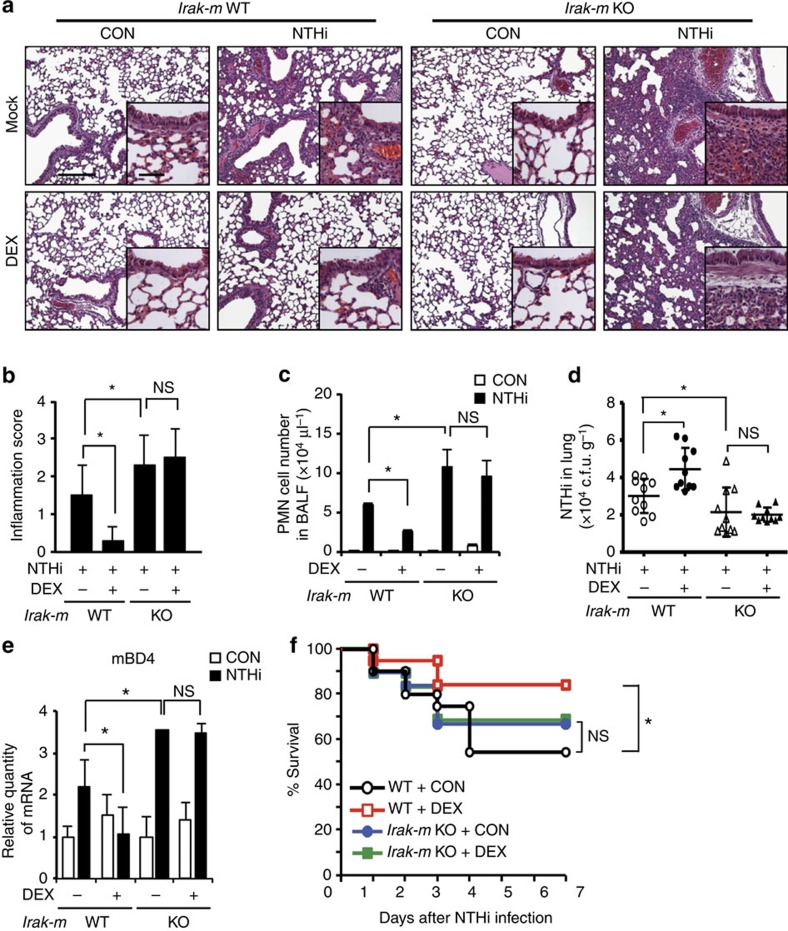
DEX suppresses NTHi-induced innate inflammatory response in *Irak-m*^*+/+*^ but not in *Irak-m*^−/−^ mice. (**a**–**e**) WT and IRAK-M-deficient mice were inoculated with DEX (1 mg kg^−1^, intraperitoneally) for 2 h, followed by intratracheal inoculation with NTHi (1 × 10^7^ c.f.u. per lung) for 24 h. (**a**) Haematoxylin and eosin (H&E) staining of lung tissues from mice (Scale bar, 200 μm, magnification, × 100 in large frame; Scale bar 50 μm, magnification, × 400 in inserted frame). (**b**) Blinded histopathologic scoring of lung inflammation was performed on H&E-stained lung sections in a grade 0–3. (**c**) The number of PMN cells from BAL fluid was counted using a haemocytometer under the microscope. (**d**) Bacterial loads (c.f.u.) in lung homogenates. (**e**) The mRNA expression of mBD4 in lung was measured by qPCR (**f**) WT and IRAK-M-deficient mice were inoculated with DEX (1 mg kg^−1^, intraperitoneally) for 2 h, followed by intratracheal inoculation with lethal dose of NTHi (5 × 10^8^ c.f.u. per lung) for 7 days. Survival rate was monitored for indicated days. *P* values were determined by Kaplan–Meier survival analysis with GraphPad Prism 5.0. Data in **b**=8; **c**,**e**, *n*=3; **d**=10, **f**=20 are mean±s.d. *P<0.05, NS, non-significant; *t-*test.

To determine if DEX suppresses NTHi-induced inflammatory response via the induction of IRAK-M expression, we compared the inhibitory effect of DEX on NTHi-induced innate inflammatory response in WT (*Irak-m*^+/+^) with IRAK-M-deficient (*Irak-m*^−/−^) mice. We observed that DEX markedly suppressed the NTHi-induced mRNA expression of key proinflammatory mediators including TNF-α, IL-1β, IL-6, MIP2, CXCL5/LIX, CXCL10 and CCL5, at the mRNA level, in *Irak-m*^+/+^ but not in *Irak-m*^−/−^ mice (Fig. 3a). Enzyme-linked immunosorbent assay analysis confirmed that DEX failed to markedly suppress the NTHi-induced expression of these proinflammatory mediators at the protein level in the bronchoalveolar lavage (BAL) fluid of *Irak-m*^−/−^ mice as compared with *Irak-m*^+/+^ mice (Fig. 3b). Consistent with these results, similar results were also observed in mouse alveolar macrophages (Fig. 3c). Moreover, histopathological analysis of the lung of NTHi-infected mice showed that DEX inhibited NTHi-induced leukocyte infiltration in the peribroncheal and interstitial area and also inhibited alveolar wall injury by the inflammatory process in *Irak-m*^+/+^ but not in *Irak-m*^−/−^ mice (Fig. 4a,b). Similarly, DEX also suppressed NTHi-induced leukocytes infiltration in BAL fluid of *Irak-m*^+/+^ mice but not of *Irak-m*^−/−^ mice (Fig. 4c). Because an innate inflammatory response initiated by pulmonary epithelial cells is critical for bacterial clearance, we further evaluated the effects of DEX on bacterial clearance in the lungs of *Irak-m*^−/−^ mice compared with *Irak-m*^+/+^ mice. DEX treatment increased the colony-formation unit (c.f.u.) of NTHi in the lung of *Irak-m*^*+/+*^ mice, but not in the lung of *Irak-m*^−/−^ mice (Fig. 4d). Consistent with the result of bacterial clearance, DEX also inhibited the NTHi-induced expression of mouse β-defensin 4, an orthologue of human BD2, in *Irak-m*^*+/+*^ mice, but not in *Irak-m*^−/−^ mice (Fig. 4e). Because overactive inflammatory responses contribute significantly to the increased morbidity and mortality in patients with NTHi infections^25^,^26^, we next evaluated the contribution of IRAK-M in mortality induced by NTHi by infecting mice with a lethal dose of NTHi (5 × 10^8^ c.f.u.) and monitoring survival rate for 7 days. As shown in Fig. 4f, we observed ~50% mortality in *Irak-m*^*+/+*^ mice 7 days after infection. We found no statistically significant difference in survival rate between DEX-untreated *Irak-m*^*+/+*^ and *Irak-m*^−/−^ mice (*P*=0.362). Notably, the survival rate was significantly improved by DEX in *Irak-m*^*+/+*^ but not in *Irak-m*^−/−^ mice (Fig. 4f). Collectively, our data suggest that DEX suppressed bacteria-induced innate inflammatory response and improved survival via the upregulation of IRAK-M expression.

### IKKβ and GR mediate synergistic induction of IRAK-M

**Figure 5:**
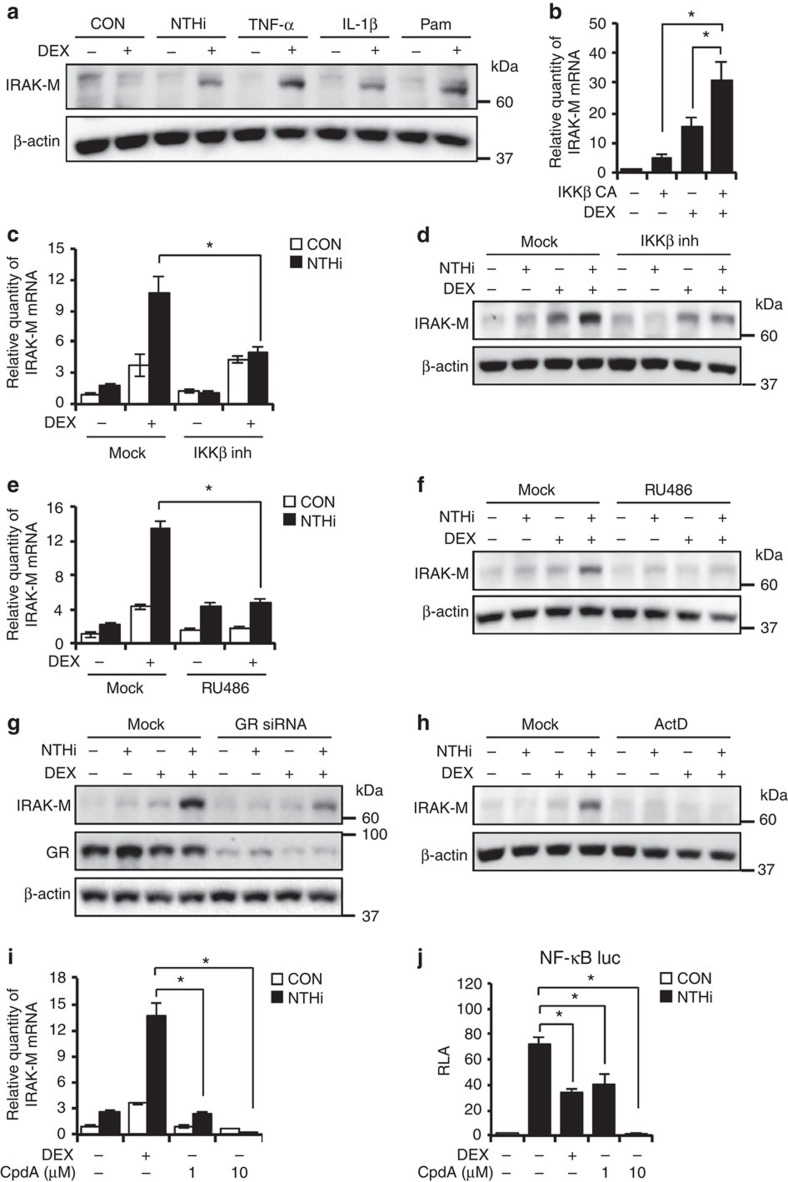
DEX synergistically enhances the NTHi-induced IRAK-M expression via IKKβ and GR. (**a**) Immunoblot shows that IRAK-M protein expression in BEAS-2B cells treated with DEX (100 nM) and NTHi, TNF-α (10 ng ml^−1^), IL-1β (1 ng ml^−1^) or Pam3CSK4 (1 μg ml^−1^). (**b**) IRAK-M mRNA expression assessed by qPCR in A549 cells transfected with constitutive active form of IKKβ (IKKβ CA) with DEX (100 nM). (**c**–**f**), IRAK-M expression in BEAS-2B cells treated with DEX (100 nM) and NTHi, IKKβ inhibitor (1 μM) (**c**,**d**) or RU486 (1 μM; **e**,**f**). (**g**) Immunoblot shows IRAK-M protein expression in BEAS-2B cells transfected with siRNA GR. (**h**) BEAS-2B cells were treated with NTHi, DEX (100 nM) and actinomycin D, ActD (5 μg ml^−1^), followed by immunoblot. (**i**) The expression of IRAK-M mRNA in BEAS-2B cells treated with DEX (100 nM) and Compound A, CpdA (1 or 10 μM) followed by NTHi stimulation for 5 h. (**j**) Relative luciferase activity (RLA) of NF-κB is measured by luciferase assay. BEAS-2B cells transfected with NF-κB luciferase plasmid were treated with CpdA and NTHi. Data (**b**,**c**,**e**,**i**,**j**, *n*=3) are mean±s.d. *P<0.05, NS; *t-*test.

Having shown that GCs suppress NTHi-induced inflammatory response via the induction of IRAK-M, the mechanism underlying the regulation of IRAK-M expression still remains unknown. Because NTHi is recognized by TLR2 (refs 27, 28, 29), we sought to first evaluate the generalizability of our findings by determining if DEX also enhances the induction of IRAK-M expression by TLR-dependent and -independent inflammatory stimuli. GCs enhanced upregulation of IRAK-M by TNFα, IL-1β and Pam3CSK4 in lung epithelial cells (Fig. 5a). Because all of these stimuli that induce IRAK-M, including NTHi, are known to induce inflammatory response via IKKβ^27^,^28^,^29^, we thus examined if DEX synergistically enhances upregulation of IRAK-M induced by activating IKKβ signalling through the overexpression of a constitutively active form of IKKβ (IKKβ CA). DEX indeed synergistically enhanced the induction of IRAK-M by the direct activation of IKKβ signalling (Fig. 5b). We further determined if IKKβ mediates the synergistic upregulation of IRAK-M by DEX and NTHi. Inhibition of IKKβ using specific inhibitor significantly suppressed the synergistic upregulation of IRAK-M expression at both mRNA and protein levels by DEX and NTHi (Fig. 5c,d), suggesting the requirement of IKKβ signalling in mediating the synergistic induction of IRAK-M.

Because GR is crucial for GC-mediated biological effects^5^,^6^, we next assessed the role of GR using RU486 (mifepristone), a GR antagonist. RU486 significantly inhibited the synergistic upregulation of IRAK-M expression at both mRNA and protein levels by DEX and NTHi in BEAS-2B and A549 cells (Fig. 5e,f and Supplementary Fig. 3a). Moreover, GR depletion using siRNA also markedly inhibited the synergistic upregulation of IRAK-M by DEX and NTHi (Fig. 5g and Supplementary Fig. 3b), indicating that GR mediates synergistic induction of IRAK-M. GR is known to regulate gene expression by influencing transcription or mRNA stability^30^,^31^. Interestingly, the mRNA stability of IRAK-M remained largely unchanged by NTHi or DEX (Supplementary Fig. 4). Consistent with this result, actinomycin D, a transcriptional inhibitor, abrogated the synergistic induction of IRAK-M protein expression induced by DEX and NTHi (Fig. 5h). These data suggest that DEX and NTHi are unable to induce IRAK-M expression in the absence of the on-going transcription.

We sought to further elucidate the molecular mechanism underlying the synergistic induction of IRAK-M by DEX and NTHi. DEX is known to exert its transcriptional activity via the induction of the ligand-dependent dimerization of GR and the subsequent binding of the dimerized GR to GRE in the gene regulatory region of the driven genes^4^,^5^,^6^. We thus first determined if the ligand-induced binding of GR dimer to the GRE in the promoter region of IRAK-M is involved in the synergistic induction of IRAK-M transcription by using a non-steroidal GR-monomer-favouring compound, compound A (CpdA). Unlike DEX, CpdA exhibits no effect on GRE-driven gene transcription^32^. We found that CpdA failed to enhance NTHi-induced expression of IRAK-M (Fig. 5i). In contrast, it still significantly inhibited the NTHi-induced NF-κB activity (Fig. 5j). These data thus suggest that the binding of dimerized GR to GRE is required for the synergistic induction of IRAK-M transcription by DEX and NTHi.

### GR and p65 synergistically bind to IRAK-M promoter

**Figure 6:**
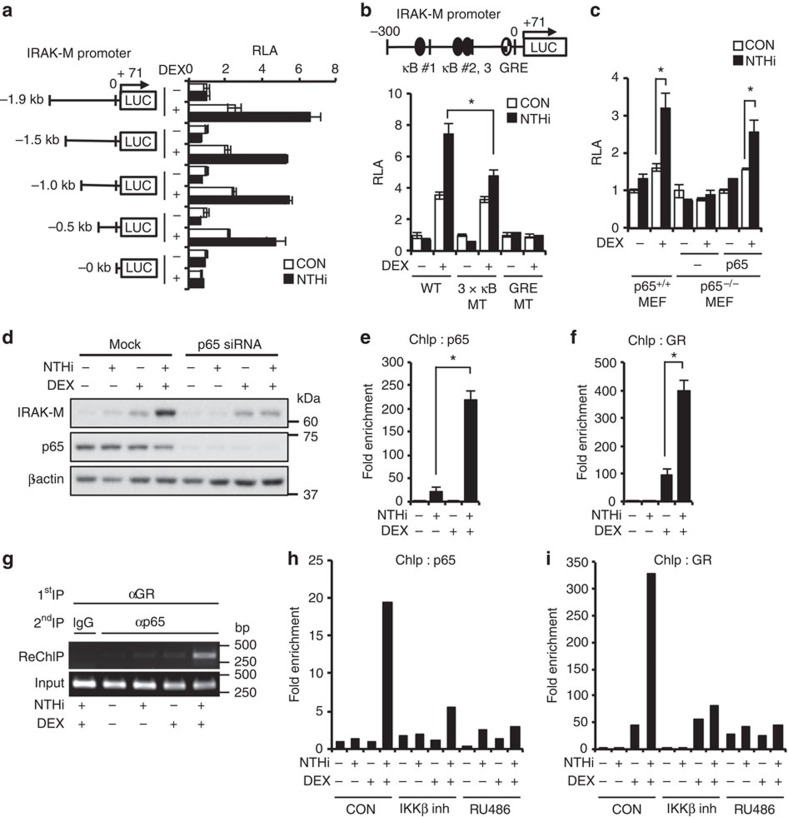
DEX and NTHi synergistically induce IRAK-M transcription via inducing the binding of p65 and GR to IRAK-M promoter. (**a**–**c**) Relative luciferase activity (RLA) was measured after the transfection of IRAK-M promoter in pGL3 basic vector and treated with DEX (100 nM) and NTHi in BEAS-2B cells (**a**,**b**). (**c**) MEF cells were transfected with IRAK-M-luc (−500 bp), followed by DEX (1 μM) and NTHi stimulation. (**d**) Immunoblot shows IRAK-M protein expression in BEAS-2B cells transfected with siRNA p65. (**e**,**f**) ChIP assay in BEAS-2B cells treated with DEX and NTHi for 1 h. IRAK-M promoter regions from −38 to +39 bp for GRE, from −231 to −99 bp for κB site were amplified by qPCR. (**g**) Re-ChIP assay (detailed in Methods). PCR products were detected in agarose gel. (**h**,**i**) ChIP assay in BEAS-2B cells treated with IKKβ inhibitor (1 μM), RU486 (1 μM), DEX (100 nM) and NTHi. Data (**a**–**c**, *n*=3; **e**,**f**, *n*=2) are mean±s.d. *P<0.05; *t-*test.

We next sought to define the *cis*-acting DNA elements critical for the synergistic induction of IRAK-M. We constructed a series of deletion mutants in the upstream region of IRAK-M promoter, inserted them to a pGL3 basic vector and measured their luciferase activity. As shown in Fig. 6a, the key *cis*-acting elements critical for mediating the synergistic induction of IRAK-M transcription resided within the promoter region from −500 to +71 base pair (bp). Further *in silico* sequence analysis revealed the existence of three putative NF-κB-binding sites (κB site) and one GRE in the IRAK-M promoter region from −300 to +71 bp (Supplementary Fig. 5). Interestingly, mutation of this GRE and these three κB sites markedly inhibited the synergistic activation of IRAK-M promoter by DEX and NTHi (Fig. 6b), thereby further demonstrating the requirement of NF-κB and GR. Since p65 is a key subunit of NF-κB^33^, we next determined if p65 is critically involved in this synergistic induction of IRAK-M. We observed no synergistic induction of IRAK-M in p65-deficient mouse embryonic fibroblasts (MEFs) treated with NTHi and DEX as compared with WT MEFs, and the reconstitution of p65-deficient MEFs with WT p65 plasmid restored their responsiveness to NTHi and DEX (Fig. 6c). Consistent with these results, p65 knockdown by siRNA inhibited the synergistic induction of IRAK-M expression at the protein level in BEAS-2B cells (Fig. 6d and Supplementary Fig. 6a) and DEX also synergistically enhanced IRAK-M expression induced by expressing WT p65 in BEAS-2B cells (Supplementary Fig. 6b). Taken together, our data suggest that both p65 and GR are required for mediating the synergistic induction of IRAK-M transcription by DEX and NTHi.

**Figure 7:**
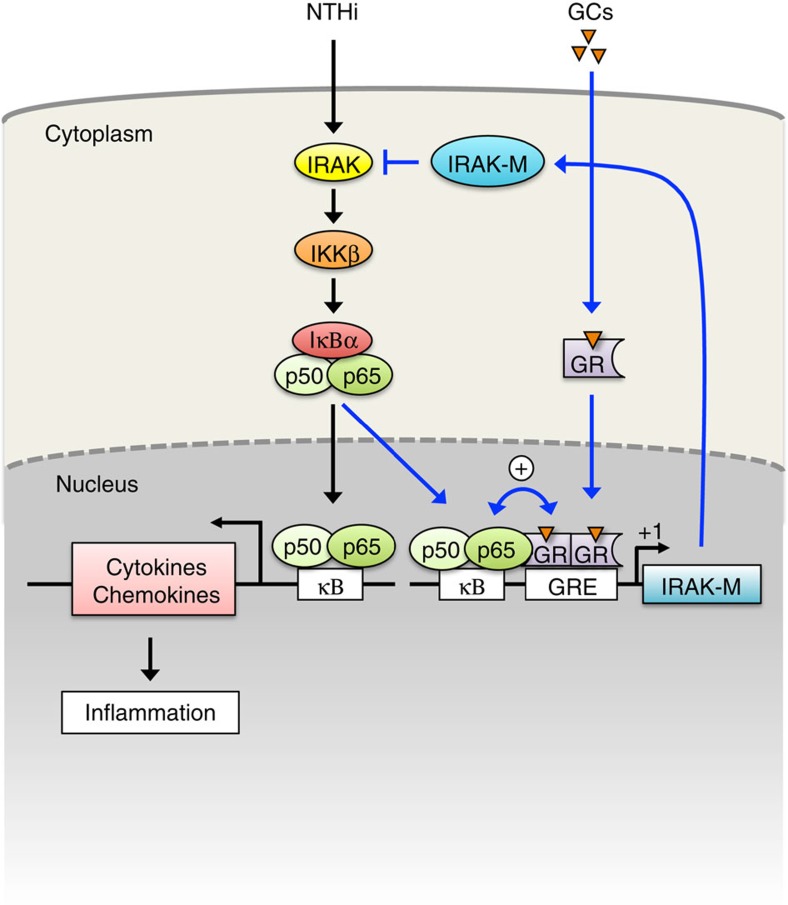
A schematic representation of suppression of inflammation by GCs via the upregulation of IRAK-M expression.

We next determined if DEX synergizes with NTHi to induce IRAK-M transcription via synergistically inducing the binding of p65 and GR to IRAK-M promoter region (−300 bp to +71 bp) containing three κB sites and GRE. Quantitative analyses using chromatin immunoprecipitation (ChIP) assays revealed that DEX and NTHi synergistically enhanced the binding of both p65 and GR to IRAK-M promoter (Fig. 6e,f). In addition, Re-ChIP assay^34^ revealed that DEX and NTHi induced the interaction of p65 with GR in the context of chromatin (Fig. 6g). Moreover, the synergistic induction of the binding of p65 and GR to IRAK-M promoter induced by NTHi and DEX was significantly inhibited by RU486 and IKKβ inhibitor, respectively (Fig. 6h,i). Together these data suggest that DEX synergizes with NTHi to induce the binding of both GR and p65 to IRAK-M promoter, which, in turn, leads to the induction of IRAK-M transcription in a synergistic manner (Fig. 7).

## Discussion

On infection, an innate inflammatory response can be activated through a TLR. The adaptor proteins IRAK1 and IRAK4 (IRAK1/4) and MyD88 mediate all TLR signalling, except TLR3, in triggering inflammation. It has been demonstrated that MyD88 and IRAK1/4 are critical in activating an inflammatory response in mice^12^,^35^ as well as in humans^11^,^36^. Since GCs have a potent anti-inflammatory effect for multiple usages in a clinical setting, we hypothesized that GCs could target central bottleneck proteins such as MyD88 or IRAK1/4 to tightly regulate inflammatory responses. Here we show that GCs synergistically enhance bacteria-induced IRAK-M, a critical negative regulator of IRAK1/4, *in vitro* and *in vivo*. We found that NTHi and GCs cooperatively induced recruitment of p65 and GR to the IRAK-M promoter. In addition, the inhibitory effect of DEX on NTHi-induced inflammatory response is significantly attenuated in mice lacking IRAK-M. Thus, we propose that IRAK-M is a novel functional target of GCs for suppression of bacteria-induced inflammation.

In the present study, we provided experimental evidence for a novel anti-inflammatory mechanisms by which GCs suppress inflammation via the cooperative upregulation of IRAK-M by the GR and NF-κB. This finding may be of particular significance as it contradicts a widespread assumption that GCs exert their anti-inflammatory effects principally by antagonizing NF-κB activity^4^,^5^. If extended to other models of inflammatory diseases, our study may have significant translational implications for design of novel anti-inflammatory agents. Previous studies have predominantly focused on understanding the GC-mediated anti-inflammatory actions through the inhibition of positive regulators such as NF-κB and AP-1. Although it has been shown that GCs suppress inflammation by targeting more distal regulators such as IκBα or MKP-1 (refs 37, 38, 39), the role of GCs in regulating more proximal and central regulators of inflammation, such as the key bottleneck proteins MyD88 and IRAK1/4, remains unknown. Recently, systematic approaches revealed that MyD88 is a non-redundant core element^40^,^41^. Owing to GCs’ prominence among anti-inflammatory agents, it is logical that GCs may target MyD88-IRAK1/4 to tightly control inflammation. Thus, our study provides novel insight into the tight regulation of MyD88 and IRAK1/4 via modulating the endogenous inhibitor IRAK-M by GCs.

GR has been shown to suppress inflammatory responses via the inhibition of p65-induced transcription^4^,^37^,^42^. A recent genome-wide study revealed that GR and p65 mutually enhance their binding to a specific promoter^43^. Thus, the regulation of p65- and GR-associated transcription is likely gene specific. Despite previous studies showing that the expression of IRAK-M is induced by inflammatory stimuli in macrophages^22^,^44^,^45^,^46^,^47^ and in osteoblasts^48^, the precise molecular mechanism of IRAK-M regulation at the transcriptional level remains to be understood. We demonstrated that the co-treatment of cells with both NTHi and GCs leads to the synergistic binding of p65 and GR to the IRAK-M promoter. Interestingly, NTHi- and GR-induced binding of p65 to the IRAK-M promoter is inhibited by the GR antagonist, RU486 (Fig. 6h). Conversely, we also observed a decrease in GR binding in the presence of IKKβ inhibitor (Fig. 6i). Consistent with previous studies, our data suggest that both p65 and GR synergize with each other to bind to the IRAK-M promoter. These data suggest a novel regulatory mechanism for IRAK-M promoter activity. Future studies investigating how the p65-GR complex activates IRAK-M transcription may lead to a better understanding of the elaborate interplay between bacteria and GCs.

Initially, IRAK-M expression was predominantly characterized in monocytes/macrophages^16^,^20^,^21^. Recently it has been shown that during respiratory infections, IRAK-M is expressed and induced, not only in alveolar macrophages, but also in respiratory epithelial cells^22^,^49^,^50^. In the present study, we found that GCs synergize with NTHi to induce IRAK-M expression in both respiratory epithelial cells and alveolar macrophages *in vitro* and *in vivo*. Importantly, GCs are unable to suppress the NTHi-induced upregulation of proinflammatory mediators in IRAK-M-depleted lung epithelial cells as well as IRAK-M-deficient alveolar macrophages. Our findings are in line with previous studies demonstrating the expression of IRAK-M in non-myeloid cells including epithelial cells. Thus, it is likely that IRAK-M expressed in both respiratory epithelial cells and macrophages are involved in mediating the ability of GCs to suppress inflammation induced by the respiratory bacterial pathogen, NTHi. It should be noted that our study did not exclude the involvement of other cell types. Future studies using conditional knockout mice with tissue-/cell type-specific deletion of IRAK-M may help determine the contribution of cell-specific induction of IRAK-M expression.

IRAK-M has been shown to act as a critical negative regulator for inflammatory responses. In line with previous findings in pneumonia models^15^,^16^,^17^,^18^,^19^,^20^,^51^, we observed elevated inflammatory responses in the lung of IRAK-M-deficient mice compared with WT mice. We found that GCs suppressed the NTHi-induced inflammation and significantly improved the survival rate in WT mice but not in IRAK-M-deficient mice (Fig. 4f). Interestingly, no statistically significant difference in survival rate was found between DEX-untreated WT and IRAK-M-deficient mice although IRAK-M-deficient mice show a trend of better survival. Our finding is consistent with a recent study showing a reduced lethality in IRAK-M-deficient mice after infection with *S. pneumonia* via the airway^20^. Given that the markers of inflammation are indeed elevated in IRAK-M-deficient mice, it is unclear why IRAK-M-deficient mice did not show an increased mortality. Although the current study does not provide a clear explanation for this apparent anomaly, our preliminary finding on the negative role of IRAK-M in bacteria-induced mucus production may provide possible explanations for this difference. Recently, there is an increasing evidence suggesting that the host has the ability to reduce the tissue damage caused directly by both pathogens and immunopathology through the enhancement of tolerance mechanisms, thus improving host survival^52^. IRAK-M may act as a negative regulator for NTHi-induced host tolerance. Indeed, our preliminary studies revealed that IRAK-M is also a negative regulator for bacteria-induced mucus production, an important mucosal defense mechanism for protecting the host from inflammation- and pathogen-induced tissue damage. Thus, IRAK-M-deficiency results in, not only overactive inflammation, but also overproduced mucus, which are apparently counteractive. This disparity may explain why IRAK-M-deficient mice did not show an increased mortality. Future studies using mice deficient in mucus production may help to further address this question.

In conclusion, our studies unveil a novel mechanism by which GCs suppress bacteria-induced innate immune and inflammatory responses by upregulating IRAK-M. Our study provides new insights into the previously unidentified role of GCs in suppressing inflammation by targeting the central bottleneck proteins MyD88 and IRAK1/4. It may also lead to the development of new therapeutic strategies to control overactive inflammation.

## Methods

### Reagents and antibodies

DEX, Mifepristone (RU486) and Actinomycin D were purchased from Sigma-Aldrich. Compound A was purchased from Enzo Life Science. IKK2 inhibitor IV was purchased from EMD Millipore. Antibodies: GR (sc-8992), p65 (sc-8008, sc-372, sc-109), IRAK-M (sc-100389), α-Tubulin (sc-69969), β-actin (sc-8432), F4/80 (sc-26642), E-cadherin (sc-31020), Donkey anti-rabbit IgG-FITC (sc-2090) and bovine anti-goat IgG-TR (sc-2786) were purchased from Santa Cruz Biotechnology, IRAK-M (#2355) from ProSci, anti-rabbit HRP-linked antibody (#7074) and anti-mouse HRP-linked antibody (#7076) were from Cell Signaling. siRNAs; GR (cat# L-003424-00-0005, ON-TARGET plus Human NR3C1 (2908)-SMARTpool, p65 (cat# L-003533-00-0005, ON-TARGET plus Human RELA (5970) - SMARTpool) and control siRNA (cat# D-001810-10-05) were from Thermo Scientific Dharmacon, IRAK-M (IRAK3 (ID 11213) cat# SR307690 and control siRNA (cat# SR30004) were from OriGene.

### Mice and animal experiments

*Irak-m*^−/−^ mice have been described previously^16^, and age-matched (8–9 weeks old) male C57BL/6 J mice were used as WT controls. For investigation of the NTHi-induced inflammation in mice, anaesthetized mice were intratracheally inoculated with NTHi at a concentration from 1 × 10^7^ to 5 × 10^8^ c.f.u. per mouse and saline was inoculated as control. The inoculated mice were then killed after NTHi inoculation. For PMN analysis, BAL fluid was collected by cannulating the trachea with sterilized PBS in mice followed by staining with Diff-Quik staining system (modified Giemsa staining). For isolation of macrophages, we collected BAL fluid with 3 ml sterilized PBS. After centrifuge the BAL fluid, macrophages were purified by percoll gradient preparation (Amersham Pharmacia Inc.). For inhibition study, mice were pretreated with DEX (1 mg kg^−1^) intraperitoneally 2 h before NTHi inoculation. All animal experiments were approved by the Institutional Animal Care and Use Committee (IACUC) at Georgia State University.

### Histology and immunostaining

For histological analysis, formalin-fixed paraffin-embedded lung tissues were sectioned (4 μm) and then stained with haematoxylin and eosin to visualize inflammatory responses and pathological changes in the lung. The stained sections were then imaged and recorded under light systems (AxioVert 40 CFL, AxioCam MRC, and AxioVision LE Image system, Carl Zeiss). The detection of IRAK-M protein was performed using rabbit anti-IRAK-M (ProSci, 2 μg ml^−1^ for Immunohistochemistry, 10 μg ml^−1^ for Immunofluorescence) and Donkey anti-rabbit-FITC (Santa Cruz Biotechnology, 5 μg ml^−1^) in the paraffin section of mouse lung tissue. Blocking IRAK-M peptide (ProSci, cat# 2355 P, 25 μg ml^−1^) was used for the negative control experiments. Epithelial cells and macrophages were recognized by antibodies of anti-E-cadherin (Santa Cruz Biotechnology, 10 μg ml^−1^) and anti-F4/80 (Santa Cruz Biotechnology, 10 μg ml^−1^), respectively and followed by bovine anti-goat TR (Santa Cruz Biotechnology, 8 μg ml^−1^) incubation. Inflammation score in haematoxylin and eosin staining (Grade; 0 to 3) and IRAK-M protein expression intensity score in immunostaining (Grade; 0 to 4) were validated in a blinded fashion^53^,^54^,^55^,^56^.

### Bacterial culture

NTHi strain 12 (also known as R2846) used in this study was a clinically isolated strain that was kindly provided by H. Faden (Children’s Hospital of Buffalo, State University of New York, Buffalo, NY)^57^. NTHi were grown on chocolate agar plate at 37 °C in an atmosphere of 5% CO_2_ overnight and inoculated in brain heart infusion broth supplemented with 3.5 μg ml^−1^ NAD and haemoglobin (BD Biosciences). After overnight incubation, bacteria were subcultured into fresh brain heart infusion and the log phase NTHi, monitored by measurement of optical density (OD_600_) value, was washed and suspended in DMEM for *in vitro* cell experiments and in isotonic saline for *in vivo* animal experiments.

### Cell culture

All media described below were supplemented with 10% fetal bovine serum (Sigma-Aldrich). Human airway epithelial A549 cells were maintained in F-12 K media (Gibco), BEAS-2B cells in RPMI 1640 media (Gibco). BEAS-2B cells stably expressing human IRAK-M were obtained by plasmid transfection following geneticin selection (300 μg ml^−1^). Human primary bronchial epithelial (Lonza) cells were maintained in BEGM (bronchial epithelial growth media) supplemented with BEGM SingleQuots. Human peripheral blood CD14+ monocytes (Lonza) were cultured in RPMI 1640 media (Gibco) containing 1 mM pyruvate and GM-CSF (50 ng ml^−1^; R&D systems). MEF cells were obtained from E13 embryos and maintained in DMEM (Corning Cellgro). p65^−/−^ MEFs reconstituted with p65 WT were cultured in DMEM (Corning Cellgro) containing puromycin (1.5 μg ml^−1^). All cells were cultured in a humidified atmosphere of 5% CO_2_ at 37 °C.

### Real-time quantitative RT–PCR analysis

Total RNA was isolated with TRIzol reagent (Life Technologies) by following the manufacturer’s instruction. The reverse transcription reaction was performed by using 1 μg of RNA in 25 μl of reaction buffer of TaqMan reverse transcription reagents (Applied Biosystems) and run under the following protocol: 25 °C for 10 min, 42 °C for 1 h and 95 °C for 5 min (refs 29, 58, 59, 60). PCR was performed by using Fast SYBR Green Master Mix (Life Technologies). In brief, the reactions were performed in triplicate containing 2 × Universal Master Mix, 1 μl of template cDNA, 400 nM primers in a final volume of 12.5 μl and they were analysed in a 96-well optical reaction plate (Applied Biosystems). Reactions were amplified under the following protocol: 95 °C for 20 s followed by 40 cycle of 95 °C for 3 s and 60 °C for 30 s and quantified by using StepOnePlus Real-Time PCR System and the manufacturer’s corresponding software (StepOnePlus Software v2.3; Applied Biosystems). The relative quantities of mRNAs were obtained by using the comparative Ct method and were normalized using human cyclophilin or mouse glyceraldehydes-3-phosphate dehydrogenase as an endogenous control. The primers are described in Supplementary Table 1.

### Plasmids and transfections

The expression plasmid of a constitutively active form of IKKβ (IKKβ-CA, S177E/S181E) was a gift from Dr Anjana Rao (Addgene plasmid # 11105) (ref. 61). The expression plasmid of p65 was cloned and the insert was transferred to pcDNA3.1 vector (Life Technologies) with BamHI and HindIII sites after we amplified the insert with primers shown in Supplementary Table 2. The luciferase reporter construct of NF-κB contains three copies of the NF-κB site from the IL-2 receptor (α) promoter by using the following oligonucleotides: 5′-TCGAGACGGCAGGGGAATCTCCCTCTCCG-3′ and 3′-CTGCCGTCCCCTTAGAGGGAGAGGCAGCT-5′ (refs 27, 62). Transient transfections were carried out using TransIT-LT1 reagent (Mirus) or Lipofectamine 2000 (Life Technologies) for plasmid DNA, DharmaFECT4 (Thermo Scientific) for siRNA following the manufacturer’s instruction. For cloning of human IRAK-M, we used pDONR223-IRAK3 as a template, which was a gift from Drs William Hahn and David Root (Addgene plasmid # 23627) (ref. 63). The insert was then transferred to pcDNA3.1 vector (Life Technologies) with XhoI and BamHI site after we amplified the insert with primers shown in Supplementary Table 2. For cloning of IRAK-M promoter, we used BAC clone RP11-937C6 (BACPAC Resources Center, Children’s Hospital Oakland Research Institute) as a template. PCR was performed using primers shown in Supplementary Table 2. PCR products were transferred into pGL3 basic vector (Promega) with MluI and XhoI site. The sequences were verified from at least three clones. The GRE and NF-κB mutants of IRAK-M promoter were constructed by using the QuikChange II site-directed mutagenesis kit (Agilent Technologies) with primers shown in Supplementary Table 2.

### Western blot analysis

Western blots were performed using whole-cell extracts in protein lysis buffer (20 mM Tris-HCl (pH 7.4), 50 mM NaCl, 50 mM Na_4_P_2_O_7_, 30 mM NaF, 5 μM ZnCl_2_, 2 mM Iodoacetic acid, 1% Triton-X) with freshly added 1 mM sodium orthovanadate and protease inhibitor cocktail (Sigma-Aldrich), separated on 8% SDS–polyacrylamide gel electrophoresis gels and transferred to polyvinylidene difluoride membranes. The membrane was blocked with 5% non-fat dry milk in Tris-buffered saline (TBS) containing 0.1% Tween 20 (TBS-T). The membrane was then incubated in a 1:2,000 dilution of a primary antibody in 5% bovine serum albumin–TBS-T at room temperature for 1 h or at 4 °C for 16 h. After washing three times with TBS-T, the membrane was incubated with 1:10,000 dilution of the corresponding secondary antibody in 2.5% non-fat dry milk–TBS-T at room temperature for 2 h. Respective proteins were developed by using Amersham ECL Prime Regent (GE Healthcare Biosciences) and image were obtained by ChemiDoc XRS+System. Images have been cropped for presentation. Full-size images are presented in Supplementary Figs 7–9.

### Chromatin immunoprecipitation

ChIP assay was performed with minor modifications of the previous study^42^,^64^. The cells were crosslinked by incubation with 1% formaldehyde at room temperature for 10 min, followed by incubation with 0.125 M glycine for 5 min. Cells were washed twice with ice-cold PBS and lysed with cell lysis buffer (50 mM HEPES (pH7.4), 1 mM EDTA, 85 mM KCl, 10% glycerol, 0.5% NP40, supplemented with protease inhibitor cocktail). Nuclei was collected by centrifugation at 850*g* for 5 min and suspended in Nuclei lysis buffer (50 mM Tris-HCl (pH 8.0), 2 mM EDTA, 150 mM NaCl, 5% glycerol, 1% Triton-X-100, 0.1% SDS, supplemented with protease inhibitor cocktail). The chromatin was sheared by sonication (Branson digital sonicator) to an average size 500 bp and precleared with Protein G PLUS-Agarose (Santa Cruz Biotechnology) or Dynabeads protein G (Life Technologies). The precleared chromatin was incubated with 5 μg of primary antibodies overnight at 4 °C, followed by incubation with Protein G PLUS-Agarose or Dynabeads protein G for 2 h. The immunoprecipitates were washed two times with ChIP wash buffer I (20 mM Tris-HCl (pH 8.0), 150 mM NaCl, 1% Triton-X-100, 0.1% SDS, 2 mM EDTA), two times with ChIP wash buffer II (20 mM Tris-HCl (pH 8.0), 500 mM NaCl, 1% Triton-X-100, 0.1% SDS, 2 mM EDTA), one time with ChIP wash buffer III (20 m Tris-HCl (pH 8.0), 150 mM NaCl, 500 mM LiCl, 1% NP40, 1% deoxycholate, 1 mM EDTA) and two times with TE buffer (10 mM Tris-HCl (pH 8.0), 1 mM EDTA). The precipitated chromatin complexes were eluted in elution buffer (1% SDS, 0.1 M NaHCO_3_) at room temperature for 30 min with vortex every 5 min. After reverse crosslink at 65 °C for 18 h and protein digestion with Proteinase K (Thermo Scientific) at 55 °C for 3 h, DNA was isolated using MiniElute PCR purification kit (Quiagen) with 30 μl elution with UltraPure DNAase/RNAase-Free Distilled Water (Life Technologies). PCR was performed with primers shown in Supplementary Table 2.

### Re-ChIP

Re-ChIP assay was performed as described previously^34^. In brief, crosslinking was performed using 2 mM disuccinimidyl glutarate (Thermo Scientific) for 45 min followed by 1% formaldehyde for 10 min at room temperature. After the first IP, immune complexes were eluted in Re-ChIP elution buffer (10 mM Tris-HCl (pH 8.0), 2 mM EDTA, 2% SDS, 15 mM DTT) including protease inhibitor cocktail at room temperature for 30 min. The elution was diluted 20 times with ChIP dilution buffer (16.7 mM Tris-HCl (pH 8.0), 167 mM NaCl, 1.2 mM EDTA, 1.1% Triton-X) supplemented with protease inhibitor cocktail, 100 μg ml^−1^ bovine serum albumin , 100 μg ml^−1^ salmon sperm DNA, followed by second IP. The second IP samples were washed, eluted and purified as described above ChIP procedure. PCR was performed with 1 μl purified DNA by using PrimeSTAR Max DNA polymerase (Takara) with primers IRAK-M −300 bp forward and IRAK-M 0 bp reverse shown in Supplementary Table 2.

### Statistical analysis

Data are shown as mean±s.d. Statistical analysis was assessed by *t-*test. *P<*0.05 was considered statistically significant.

## Author contributions

M.M., J.-Y.L., S.S.-M., H.X. and J.-D.L. designed the experiments and analysed the data. M.M., J.-Y.L. and S.S.-M. performed the experiments. H.K. contributed to data analysis and discussion. R.A.F. and K.S.K. contributed materials and also contributed to discussion. M.M., W.Y.W. and J.-D.L. wrote the manuscript.

## Additional information

**How to cite this article**: Miyata, M. *et al.* Glucocorticoids suppress inflammation via the upregulation of negative regulator IRAK-M. *Nat. Commun.* 6:6062 doi: 10.1038/ncomms7062 (2015).

## Supplementary Material

Supplementary Information Supplementary Figures 1-9 and Supplementary Tables 1-2.
