# Supplementary material for: Glucocorticoids suppress inflammation via the upregulation of negative regulator IRAK-M
Source: Nat Commun. 2015 Jan 14;6:6062. doi: 10.1038/ncomms7062 (PMC4309435; doi:10.1038/ncomms7062)
Supplement: Supplementary Information — Supplementary Figures 1-9 and Supplementary Tables 1-2. [file ncomms7062-s1.pdf]

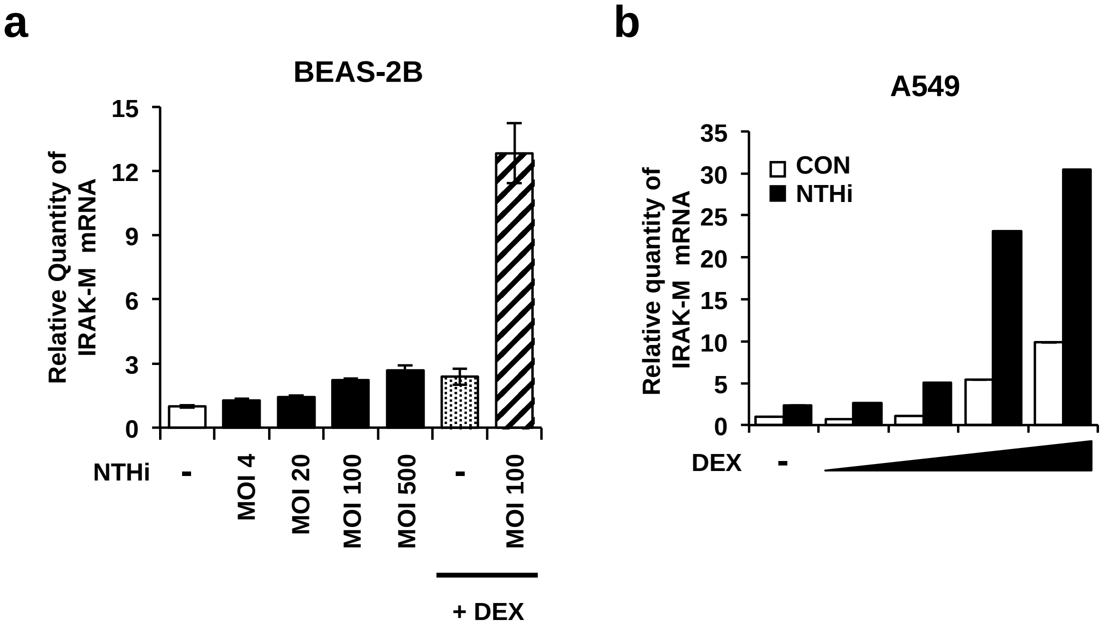

Supplementary Figure 1. DEX synergistically enhances NTHi-induced IRAK-M expression.

(**a**) IRAK-M mRNA expression in BEAS-2B cells stimulated with DEX (100 nM) for 1 h, followed by NTHi for 5 h. (**b**) IRAK-M mRNA expression in A549 cells stimulated with DEX (1, 10, 100 or 1000 nM) for 1 h, followed by NTHi for 5 h. The expression of IRAK-M mRNA was assessed by Q-PCR. Data (n = 3) are mean ± s.d.

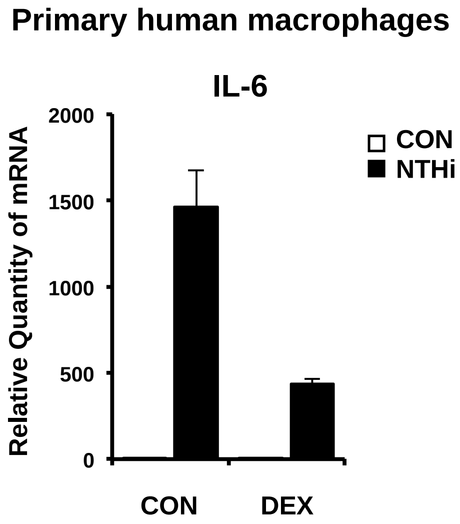

Supplementary Figure 2. DEX suppresses NTHi-induced IL-6 expression in primary human macrophages.

Human CD14$^+$ monocytes were differentiated by GM-CSF (50 ng ml^{-1}) for 7 days. The mRNA expression was assessed by Q-PCR after cells were pre-stimulated with DEX (100 nM) for 1 h, followed by NTHi for 5 h. Data (n = 3) are mean ± s.d.

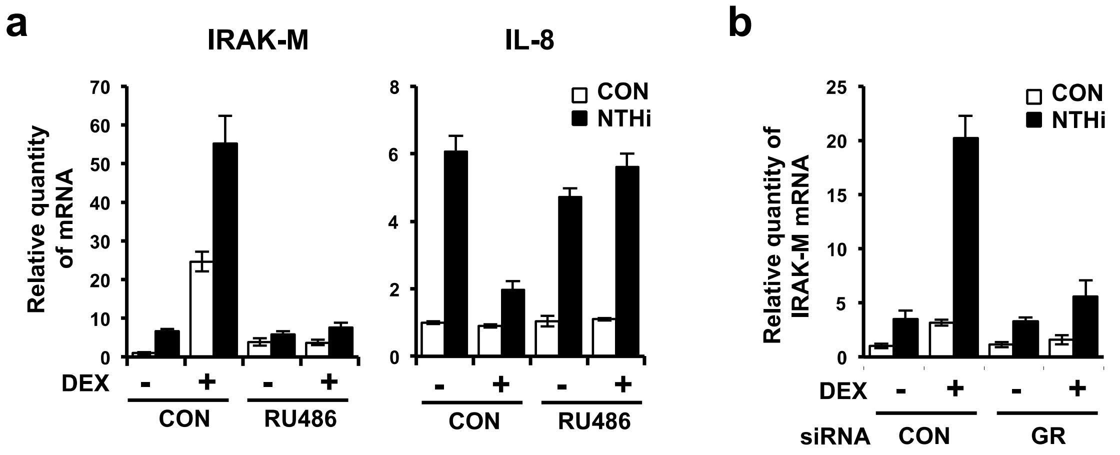

Supplementary Figure 3. DEX induces mRNA expression of IRAK-M via GR.

(**a**) A549 cells were treated with DEX (100 nM) and RU486 (1 μM) for 1 h and subjected to NTHi stimulation for 5 h. IRAK-M and IL-8 mRNA assessed by Q-PCR. (**b**) BEAS-2B cells were transfected with siRNA for 72 h, followed by stimulation with NTHi and DEX (100 nM). The expression of IRAK-M mRNA was assessed by Q-PCR. Data (n = 3) are mean ± s.d.

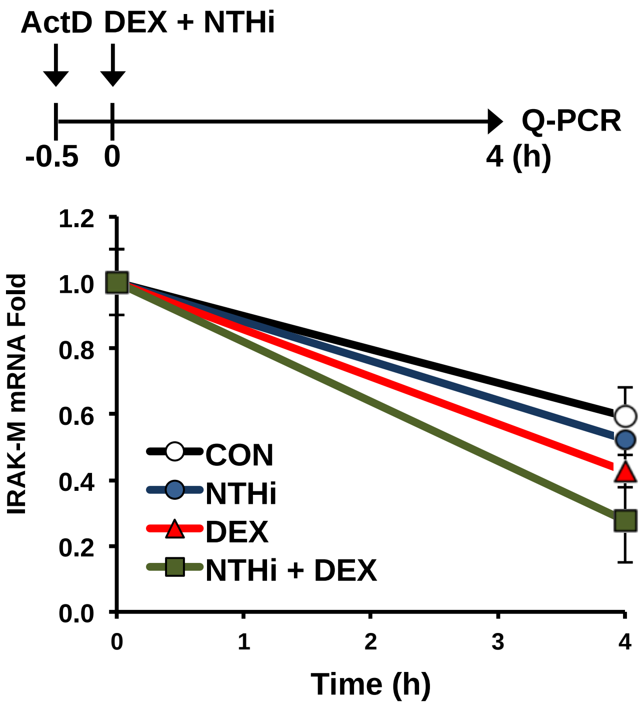

Supplementary Figure 4. mRNA stability of IRAK-M in the presence of DEX and NTHi.

BEAS-2B cells were stimulated with ActD (5 μg ml^{-1}), followed by NTHi and DEX (100 nM) for 4 h. The mRNA fold was calculated by mRNA level at 4 h/mRNA level at 0 h. Data (n = 3) are mean ± s.d.

```
-300 bp TGTAAGAAGTAATGACACCGCTAGCCGTCCACACCAGGAGACCGCCTAGCCGTGGGGCACGGTGG

        GCTCCTGGGAGCTCTGAGCTCTGGGCTTTCTCCAGTTCGCACTCTGCTTGTCTCGGCAGCTCCGT
                                 NF-κB #1
        CCCCACCGCAGAGGTGTGAAGCGCAAAGCCAGCGAAGGGAGAACCCGGGTCGGGTAACCCCCAGG
                                                  NF-κB #2          NF-κB #3
        CCTGGCCAGGCGGACGCAGGGGCATCTCGGGCGAGGCGCGCCTTGCGTCACGTGGGCACCGCCCC
                                                +1 ┌──►
        TGCAGTGACCGGAGAACGGCGTGTTCCTAGGGCTCTGCTGCCGTCGTGGAAGCAGGATTTCCGCG
                      GRE
        GTTGTGTAACGGCCTGTCGCAGGCGTGCAGGGACCTGGACTCC  +71 bp
```

Supplementary Figure 5. The sequence of human IRAK-M promoter.

The sequence is from IRAK3 ID: 11213 in NCBI database. We described IRAK3 mRNA (NM_007199.2) at position 66,189,198 as a position +1 shown with arrow.

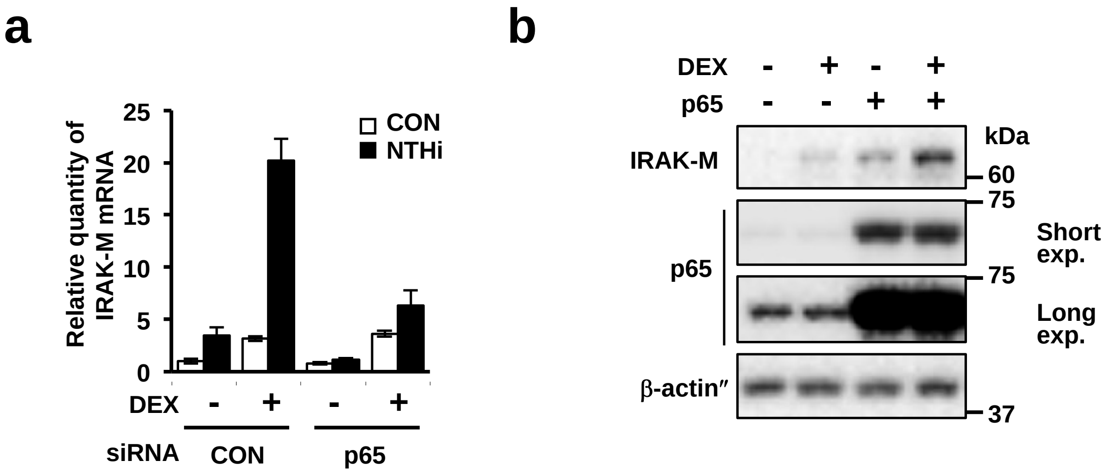

Supplementary Figure 6. p65 enhances DEX-induced IRAK-M expression.

(**a**) BEAS-2B cells were transfected with control siRNA or p65 siRNA for 72 h, followed by stimulation with NTHi and DEX (100 nM). The mRNA of IRAK-M was determined by Q-PCR. Data (n = 3) are mean ± s.d. (**b**) Immunoblot for indicated proteins in BEAS-2B cells transfected with mock or p65 for 48 h and stimulated with DEX (100 nM) for 9 h.

Fig. 1c

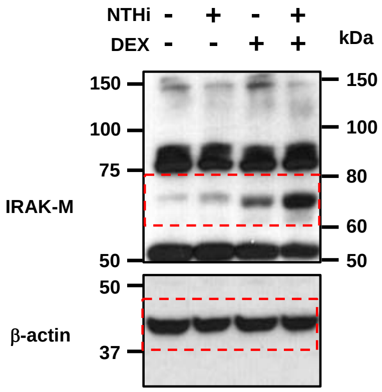

Fig. 2a

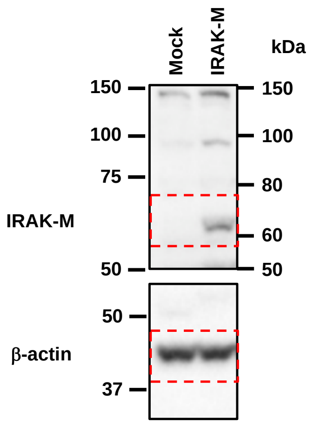

Fig. 2c

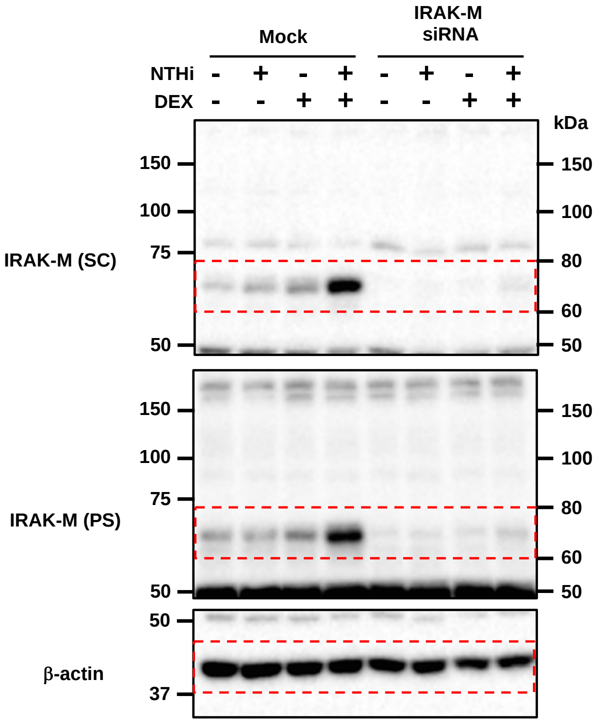

Supplementary Figure 7. Uncropped Immunoblot images with molecular weight markers shown in Fig. 1 and 2.

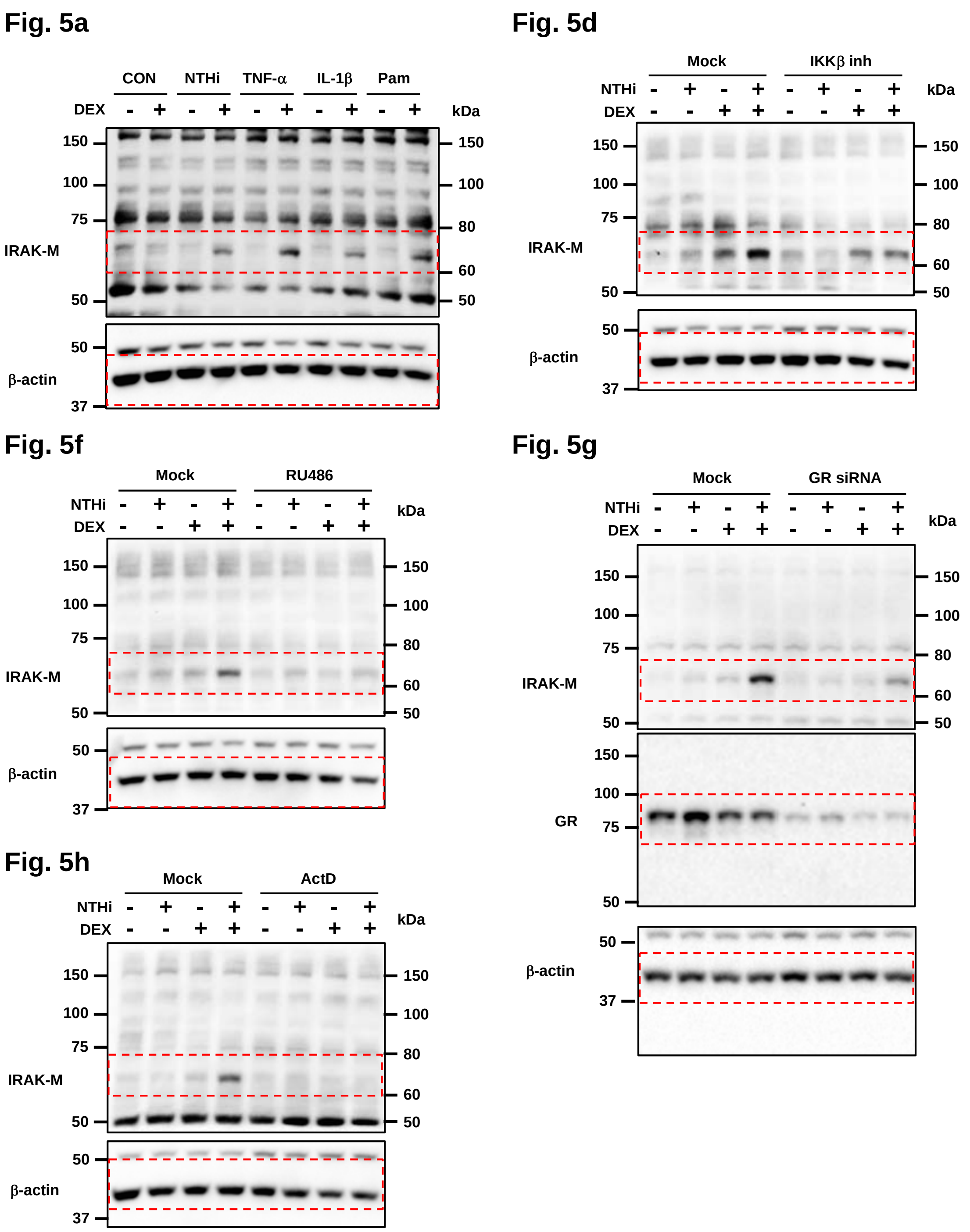

Supplementary Figure 8. Uncropped Immunoblot images with molecular weight markers shown in Fig. 5.

Fig.6d

Supplementary Figure 6

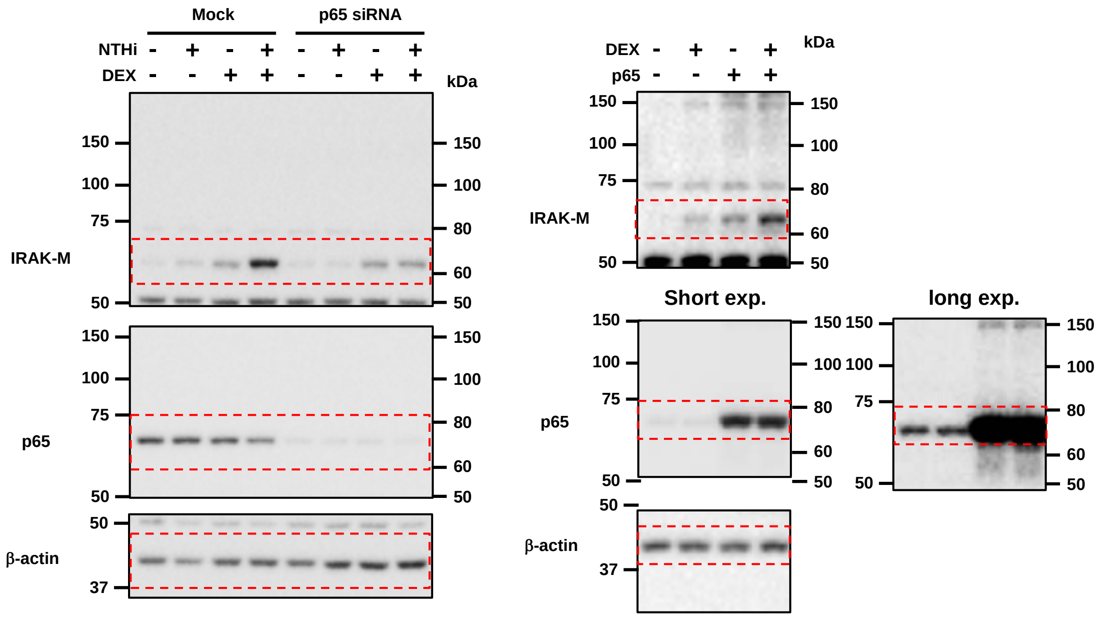

Supplementary Figure 9. Uncropped Immunoblot images with molecular weight markers shown in Fig. 6 and Supplementary Figure 6.

Supplementary Table 1. Q-PCR primers used in this study

Q-PCR Primers

Human

Gene	Primer	Sequence 5' --> 3'
Cyclophilin A	forword	CGGGTCCTGGCATCTTGT
	reverse	GCAGATGAAAAACTGGGAACCA
IL-8	forword	TCCTGATTTCTGCAGCTCTG
	reverse	GTCCACTCTCAATCACTCTCAG
IL-6	forword	AAATTCGGTACATCCTCGACGGCA
	reverse	AGTGCCTCTTTGCTGCTTTCACAC
IL-1β	forword	AAACAGATGAAGTGCTCCTTCCAGG
	reverse	CTTGTCCATGGCCACAACAACTGAC
TNFα	forword	CCCAGGCAGTCAGATCATCTT
	reverse	AGCTGCCCCTCAGCTTGA
CCL5	forword	CTACACCAGTGGCAAGTGC
	reverse	CTTTCGGGTGACAAAGACGAC
CXCL10	forword	GAAATTATTCCTGCAAGCCAATTTTG
	reverse	CCCTTCTTTTTCATTGTAGCAATG
IRAK-M	forword	CACAACGTTCAACCATGCTC
	reverse	TGTTTACTGCTGCTGCTGGT

Mouse

Gene	Primer	Sequence 5' --> 3'
Gapdh	forword	ACCCAGAAGACTGTGGATGG
	reverse	GGATGCAGGGATGATGTTCT
Mip2	forword	CCTGCCAAGGGTTGACTTCA
	reverse	TTCTGTCTGGGCGCAGTG
Il-6	forword	GAGGATACCACTCCCAACAGACC
	reverse	AAGTGCATCATCGTTGTTCATACA
Il-1β	forword	CAACCAACAAGTGATATTCTCCATG
	reverse	GATCCACACTCTCCAGCTGCA
Tnfα	forword	CATCTTCTCAAAATTCGAGTGACAA
	reverse	TGGGAGTAGACAAGGTACAACCC
Ccl5	forword	GCTGCCCTCACCATCATCCT
	reverse	GGAGTGGTGTCCGAGCCATA
Cxcl5	forword	GCTGGCATTTCTGTTGCTGTTC
	reverse	GGCAGCTTCAGCTAGATGCT
Cxcl10	forword	AGTGCTGCCGTCATTTTCTG
	reverse	TGCGAGAGGGATCCCTTGAG
Irak-m	forword	CCTGAACATAATGAAAAAGGAACAC
	reverse	ATGCTTGGTTTCGAATGTCC
BD4/Defb4	forword	CACATTTCTCCTGGTGCTGCT
	reverse	GATAATTTGGGTAAAGGCTGCAAG

Supplementary Table 2. Primers used in this study

Cloning

Gene	Primer	Sequence 5' --> 3'
Human IRAK-M	forword (XhoI)	GTACTCGAGAAATGGCGGGGAACTGTG
	reverse (BamHI)	GTAGGATCCTTATTCTTTTTTGTACTGTTCATATTCATC
Human p65	forword (BamHI)	GATGGATCCATGGACGAACTGTTC
	reverse (HindIII)	GATAAGCTTTTAGGAGCTGATCTGAC

Promoter cloning

Promoters	Primer	Sequence 5' --> 3'
-1871 IRAK-M pro	forword (MluI)	CTCACGCGTCAGCATGTTCAGGTAGTC
-1500 IRAK-M pro		CATACGCGTGGAAGGAAGGAAGGTAGGTAG
-970 IRAK-M pro		CTCACGCGTCCAAGTAGGTGTATGGAG
-500 IRAK-M pro		CTAACGCGTGACTGTGGTCCCAGCTACTC
-400 IRAK-M pro		CTAACGCGTTGGGCGACAGAGTGAGAAC
-300 IRAK-M pro		CTGACGCGTTGTAAGAAGTAATGACACCGCTAG
-200 IRAK-M pro		CTGACGCGTTTCGCACTCTGCTTGTCTC
-100 IRAK-M pro		CTTATTATATTAACGCGTTGGCCAGGCGGACGCAG
0 IRAK-M pro		GTAACGCGTCTGCCGTCGTGGAAGC
+71 IRAK-M pro	reverse (XhoI)	CTACTCGAGGGAGTCCAGGTCCCTGCAC
+0 IRAK-M pro		CTACTCGAGCAGAGCCCTAGGAACAC

Mutagenesis

Name	Primer	Sequence 5' --> 3'
IRAK-M GREmt	sense	CTGCAGTGACCGGACACAGGCGAGTTGCTAGGGCTCTGCTG
	anti-sense	CAGCAGAGCCCTAGCAACTCGCCTGTGTCCGGTCACTGCAG
IRAK-M_NF-κBmt1	sense	GAGCTCTGAGCTCTATGCTGTCTCCAGTTCGCACTCTG
	anti-sense	CAGAGTGCGAACTGGAGACAGCATAGAGCTCAGAGCTC
IRAK-M_NF-κBmt2	sense	GCAAAGCCAGCGAAATGAGAACACGGGTCGGGTAAC
	anti-sense	GTTACCCGACCCGTGTTCTCATTTCGCTGGCTTTGC
IRAK-M_NF-κBmt3	sense	GAGAACCCGGGTCATGTAACCACCAGGCCTGGCCAG
	anti-sense	CTGGCCAGGCCTGGTGGTTACATGACCCGGGTTCTC

ChIP

Name	Primer	Sequence 5' --> 3'
IRAK-M ChIP GRE	forword	CTGCAGTGACCGGAGAAC
	reverse	CGTTACACAACCGCGGAAATC
IRAK-M ChiP κB	forword	CTGGGAGCTCTGAGCTCTG
	reverse	CAGGCCTGGGGGTTACC
